# A qualitative exploration of community kitchens to reduce household food waste as a public health intervention

**DOI:** 10.1186/s12889-025-25903-2

**Published:** 2025-12-10

**Authors:** China R. Harrison, Jenna Parton, Patricia E. Jessiman, Rona Campbell, Frank de Vocht

**Affiliations:** 1 PHIRST insight, Centre for Public Health, Population Health Sciences, Bristol Medical School, University of Bristol, 9th Floor, Whitefriars, Lewins Mead, Bristol, BS12NT UK; 2 National Institute for Health Research Applied Research Collaboration West (NIHR ARC West), 9th Floor, Whitefriars, Lewins Mead, Bristol, BS1 2NT UK; 3 National Institute for Health Research, Health Protection Research Unit (HBU) in Evaluation and Behavioural Science (EBS), Population Health Sciences, Bristol Medical School, University of Bristol, Oakfield Grove, Bristol, BS8 2BN UK; 4 Leicestershire Public Health Team, Leicestershire County Council, County Hall, Glenfield, LE3 8RA UK

**Keywords:** Community kitchens, Health and wellbeing, Household food waste, Leicestershire, Process evaluation, Social interaction, Social isolation

## Abstract

**Background:**

Household food waste (HHFW) has been described as a contemporary multifactorial problem impacting the environment, economy, society and health. Community kitchens have been found to have public health benefits for social wellbeing and nutrition. However, few community kitchens have been developed and implemented with the primary aim of reducing HHFW. This research focuses on the community kitchen scheme in Leicestershire that was established to engage individuals with HHFW issues and build community capacity to deal with food waste effectively and sustainably. The aims of the research were to explore how aligned the aims of the community kitchens were to the reasons why participants attend, and the benefits experienced from participating.

**Methods:**

The research used a short survey of attendees (*n* = 33), observations of community kitchen sessions (*n* = 4) and individual semi-structured interviews with attendees (*n* = 14), volunteers (*n* = 3) and Borough Council staff (*n* = 2). Quantitative data was, analysed descriptively, and qualitative data was analysed using thematic analysis.

**Results:**

Findings suggest a gap between the reasons behind the development and implementation of the community kitchen scheme, their intended impact and the actual practices and benefits experienced by participants. Reducing HHFW was not found to be the core element, nor a reason for why people chose to attend. The community kitchens were, however, valued and considered worthwhile by participants, volunteers, and staff. In alignment with other data on the value of community kitchens, they were found to be beneficial particularly in terms of offering opportunities to socialise in an inclusive, supportive environment.

**Conclusion:**

A refocus of the community kitchens as a scheme to provide social benefits, is likely to improve the scheme’s reach and subsequent contribution to the health and wellbeing of socially isolated individuals.

**Supplementary Information:**

The online version contains supplementary material available at 10.1186/s12889-025-25903-2.

## Introduction

Cooking intervention programmes have been implemented to improve eating behaviours, nutritional status, body weight, cooking skills, financial wellbeing, and food security [1–3]. Systematic reviews suggest that such programmes can lead to favourable outcomes in both the general population [3] and specific patient populations (e.g., patients with eating disorders; [4]). However, most reported effect sizes are small to moderate in magnitude and often diminish over time. The majority of studies have also been conducted in community or clinical settings, limiting generalisability. Methodological challenges are also common, including short follow-up periods, reliance on self-reported dietary outcomes and heterogeneity in programme design and delivery. These factors constrain the ability to draw firm conclusions. Nonetheless, cooking interventions remain a promising public health strategy, with evidence linking them to improved diet quality [5–12] and reduced fast food consumption [8].

Community kitchens are cooking interventions that involve small groups of people meeting to plan, cook and share healthy and affordable meals. The main difference between community kitchens and other cooking interventions (e.g., food assistance; soup kitchens) are their collaborative participatory characteristics, and their potential to foster social skills and support [13].

Previous studies have examined the impact of community kitchens on health promotion and food security and results from such studies have suggested them to offer an effective public health strategy for improving nutrition [5–8]. For example, research has repeatedly found attendees to report improvements in their intake of nutritious and diverse food [9–12]. Community kitchens have also been suggested to influence diet of the wider family because attendees feed their families healthier food [14].

Other benefits of participating in a community kitchen programme have included decreased isolation, friendship development, mutual aid, moral support, increased self-confidence and self-esteem, and increased participation in other community events/organisations [5, 12, 14–19]. Loneliness and social isolation are growing public health concerns in the UK and globally, with a significant increase post-pandemic [20]. Loneliness has been defined as a subjective negative feeling associated with a perceived lack of a wider social network while social isolation has been defined as having a paucity of social contacts and interactions with family members, friends, or the wider community [21]. Loneliness and social isolation have been associated with a reduction in health status (e.g., cognitive decline, mental health conditions [22]) and decreased quality of life [23]. The degree to which individuals are part of communities has therefore been shown to have a powerful impact on health and wellbeing, particularly for individuals aged over 60 years [22] and individuals with disabilities, as these groups have been shown to be at a higher risk of social isolation and loneliness because they have decreased mobility and income, poor health, fewer friends and less social support [24–27]. Loneliness can also be associated with informal carers [20]. Although previous research evidence suggests community kitchens to have public health benefits, few community kitchens have been developed and implemented with the primary aim to improve public health by reducing household food waste (HHFW) and subsequent public health co-benefits.

HHFW has been described as a contemporary multifactorial problem impacting the environment, economy, society, and health [28]. Over 13 million people in the UK struggle to access enough food and numbers are increasing due to the cost-of-living crisis [29]. However, according to the most recent report by the charity Waste and Resources Action Programme (WRAP), the UK produced approximately 9.5 million tonnes of food waste in 2018, 6.6 million tonnes (70%) of which was from households [30]. There are also significant nutritional losses embedded in food waste, with more nutritional foods such as fruit and vegetables, being more likely to be wasted [31]. From an environmental health perspective food waste is associated with a waste of resources involved in the production of the food, for example, water and energy [32]. Consequently, several studies have established that better management of HHFW has the potential to lower greenhouse gas emissions and reduce the environmental footprint of food systems [33, 34] in addition to increasing food security by improving access to nutritious food [34, 35]. The issues of climate change and food security calls for increased attention to be given towards findings new and effective ways of reducing HHFW [36].

This paper is concerned with the community kitchen scheme in Leicestershire, a county in the East Midlands, England. The community kitchen scheme was initiated in 2017 by Leicestershire County Council (LCC) and a national supermarket with the aim to engage individuals from all walks of life with HHFW issues and build community capacity to reduce food waste effectively and sustainably. Originally, LCC set up three community kitchens and applied the Waste and Resources Action Programme (WRAP) (2014) and *Love Food Hate Waste* (LFHW) resources which aim to reduce the climate impact of food and drink [30]. WRAP is a UK-based non-profit organisation that works with governments, businesses, and communities to promote sustainable resource use, including reducing food waste. LFHW is a public-facing campaign developed by WRAP that aims to raise awareness and change household behaviours around food waste through practical advice and national education efforts. Both initiatives are grounded in behavioural research and national waste data, and are implemented in collaboration with local authorities, retailers, and community groups. These included: (1) meal planning; (2) understanding labelling on food; (3) using shopping lists; (4) storing and using leftovers; (5) portioning properly; and (6) food storage advice, in the community kitchens context, as part of their *Waste Less Save More* project. These focus areas reflect the most common causes of household food waste identified in the literature and by WRAP, including over-purchasing, poor storage practices, confusion over date labelling, cooking or serving too much food, and a lack of planning before shopping. By addressing these behavioural drivers directly through hands-on learning and discussion, the community kitchens aim to empower individuals to adopt more sustainable food management practices in their everyday lives. At the time of this research there were six community kitchens in Leicestershire operating on a fortnightly basis in community houses (i.e., community centres) and churches with suitable facilities such as cookers, sinks, tables and chairs and equipment to prepare, cook and store food. These were co-ordinated by volunteers and/or Borough Council staff. The initiation and development of each of the six community kitchens was funded by LCC. However, today the kitchens are financially independent.

An unpublished internal report evaluating the three original community kitchens, found them to be associated with reduced HHFW with LCC reporting participants to have reduced their HHFW by, on average, 33% over the course of a six-to-eight-week period. The aims of the current study were to qualitatively explore how aligned the aims of the community kitchens are as a public health intervention (that is a programme intended to improve the health of the public) to reduce HHFW to the reasons why participants attend the community kitchens. The second aim of the research was to gain a better understanding as to the benefits experienced from participation.

## Methods

Data collection was part of a wider mixed methods process evaluation of the Leicestershire community kitchens scheme [37, 38]. For the current descriptive research, data collection included a short survey of community kitchen attendees, observations of community kitchen sessions, and individual semi-structured qualitative interviews with community kitchen attendees, volunteers, and Leicestershire Borough Council staff. Survey data aimed to identify who was attending the community kitchens while qualitative data gathered from observations and interviews aimed to allow a more in-depth exploration of reasons for participating and benefits experienced.

Prior to all data collection activities, participants were sent a detailed participant information sheet detailing the aims of the study, information about confidentiality, anonymity and use of data collected. Signed or verbally obtained informed consent was provided by all participants prior to data collection. Ethical approval for the study was awarded by the University of Bristol’s Faculty of Health Sciences Research Ethics committee in March 2022 (ref. 10175). This study adhered to the ethical principles outlined in the Declaration of Helsinki.

### Public and Community Involvement and Engagement (PCIE)

Leicestershire County Council were involved in the development of the study protocol and design of data collection tools. The study team also undertook a two hour online public involvement workshop with previous community kitchen attendees (*n* = 2) and volunteers (*n* = 1) and individuals with no experience of community kitchens (*n* = 3). During the workshop, attendees were consulted about the participant information sheet (PIS) data collection methods (e.g., observation framework, survey questions, topic guide for interviews) and recruitment strategy. This workshop took place before recruitment began to ensure study methods and materials were relevant, inclusive, and accessible. Two individuals who attended the PCIE workshop were recruited to participate in regular study management meetings for the duration of the study.

### Sampling and recruitment

#### Participants

Eligible participants included all individuals who were involved with the community kitchens as attendees, volunteers, or staff members. Those not involved with the community kitchens were ineligible to participate.

##### Data collection 1: survey

A paper survey developed for this study was distributed on seven days throughout May June and July to all willing community kitchen attendees at the beginning of each session. It was distributed by the volunteers, Borough Council Staff or by the first author (CH). Quantitative data was gathered about attendees’ sociodemographic status (e.g., gender, age, employment) and at the end of the survey, participants were invited to leave their contact details if they were interested in participating in an interview about their experiences of participating in the community kitchen scheme (see supplementary material 1 for survey).

##### Data collection 2: observations

Four of the six community kitchens were observed once during May-June 2022 by the first author (CH). Each observation was scheduled for a day when the community kitchen was running at each of the selected locations. The four kitchens were selected based on participant characteristics (i.e., gender) and location (i.e., rural or urban). Three of the selected kitchens were gender specific (i.e., one for men only, two for women only). The other kitchen was mixed gender. All expect one of the community kitchens were in urban areas.

The researcher observed the adherence to the core elements of the sessions (i.e., cooking activities and WRAP resources), how the sessions worked, what was happening and why, what were regular and irregular activities, participant interactions with each other and the volunteers and what impact they may be having on the health and wellbeing of participants. Research was aided by an observation framework that comprised five key categories: Activities, Environment, Interactions, Objects, and Users which enabled detailed field notes to be recorded (see supplementary material 2).

##### Data collection 3: interviews

Individuals who left their contact details at the end of the survey, in addition to the volunteers and Borough Council staff who facilitated the community kitchen sessions, were contacted and invited to participate in a short interview. Attendees who did not complete the survey were also able to volunteer to participate in an interview. Semi-structured interviews were carried out face to face, by telephone or online depending on the attendees’ preferences, availability and access to facilities and resources (e.g., internet and/or computer access). The interviews were carried out in June and July 2022 by the first author (CH) and were guided by a topic guide (see supplementary material 3) which was informed by our research questions and PCIE workshop. Interviews explored the attendees’ reasons for and experiences of attending the community kitchens, reducing household food waste and the perceived benefits experienced from attending the community kitchens. Data collection and analysis were conducted iteratively, with the final sample size being guided by sufficient variation across participant characteristics (e.g., gender, age) to capture a range of perspectives and when no new insights emerged from the most recent interview data. The first author (CH) kept reflexive notes throughout the fieldwork phase of the research, and these were taken into account during data coding and analysis. These notes included reflections on interviewer assumptions, rapport with participants, and the influence of the research context on participant behaviour and data collection. Key reflections were also discussed with the research team, as they arose, and small changes were made to the research process where this was deemed necessary to improve the quality and integrity of the data collection and analysis.

Interviews lasted between 14 and 66 min (Mean 34 min). Interview topic guides were adapted so that they were applicable to intended audience (i.e., community kitchen attendees, volunteers, or Borough Council staff). Individuals who participated in the interviews were reimbursed £15 for their time.

### Data analysis

#### Survey

Data was analysed descriptively using STATA Version 17.0 [39]. Data was descriptive in nature and results for sociodemographic data are presented in percentages due to the categorical nature of the responses. Percentages vary according to the number of attendees completing each question (i.e., missing data).

#### Observations

For the observations, the researcher (CH) reviewed the field notes using a structured observation framework comprising five domains: Activities, Environment, Interactions, Objects, and Users. Data were organised and thematically coded within these categories to identify patterns relevant to the research questions. A narrative synthesis of the findings is presented in the Results section.

#### Interviews

Each of the qualitative interviews was transcribed verbatim, imported into NVivo software, and analysed followed the principles of thematic analysis [40]. Firstly, the transcripts were read several times, to gain familiarisation with the data and to note initial ideas and potential codes (e.g., social interaction and company, personal support, inclusive environment, perceived benefits). The transcripts were then examined on a line-by-line, with inductive codes generated directly from the data and deductive codes applied to the segments of the data that provided insight into the participants experiences and benefits of participating in the community kitchens (i.e., the aims of the research). Data was coded inductively by the first author (CH, a behavioural scientist) to capture participants lived experiences, while deductive attention was also paid to aspects relevant to the study aims (e.g., household food waste reduction, inclusivity). Coding was an iterative and interpretive process, with codes continuously reviewed and refined as understanding deepened. Coding was also reviewed by TJ (a qualitative public health researcher) to cross check the codes and provide alternative perspectives. In line with the reflexive approach to thematic analysis, the analysis was iterative and interpretive, with the researcher keeping reflexive notes documenting evolving assumptions, responses and interpretive shifts. For example, early reflections revealed a tendency for the focus of the community kitchens to be social rather than to reduce waste, which prompted a reconsideration of how participants narratives about support were represented, leading to a more nuanced theme around the health and wellbeing benefits of the kitchens. Reflective discussions with the wider multidisciplinary team provided alternative perspectives that supported interactive depth rather than inter-coder reliability.

Themes were developed through a recursive process of reviewing, refining and grouping codes into broader patterns that captured participants’ experiences and perceived benefits of community kitchens. The coding framework and identified themes were discussed by the multi-disciplinary research team, recognising contextual influences and different perspectives to ensure consistency, to ensure transparency, coherence and reflexivity. Reporting was informed by the COREQ checklist to strengthen methodological rigour.

Qualitative data is presented as a summary accompanied by illustrative verbatim quotations. Within illustrative quotations the use of […] indicates part of the quotation was not presented because it was not relevant, whereas (text) indicates additional text was added for clarity (i.e., readability, comprehensibility). Grammatical errors were corrected and idioms (e.g., ‘like, ‘you know,’ ‘kind of’) removed. Verbatim quotations were labelled according to whether they were from a volunteer or staff (S/V) or participant (P) and accompanied by a participant number.

## Results

### Survey

A total of 45 individuals who attend the six community kitchens in Leicestershire were asked to complete the survey. Of the 45 individuals a total of 33 completed the survey (73.3% response rate). Results for attendees’ sociodemographic characteristics are shown in Table 1.

**Table 1:** Sociodemographic characteristics for community kitchen attendees^1^

	Community kitchen attendees (*N* = 33)
**Age**	**N (%)**
25–34	2 (6.1)
35–44	0 (0.0)
45–54	5 (15.2)
55–64	5 (15.2)
65–74	10 (30.3)
75–84	9 (27.3)
85+	1 (3.0)
Missing	1 (3.0)
**Employment**	
Employed	2 (6.1)
Unemployed	1 (3.0)
Retired	13 (39.4)
Unable to work (physical)	3 (9.1)
Unable to work (mental)	2 (6.1)
Home maker	2 (6.1)
Other^2^	7 (21.2)
Missing	3 (9.1)
**Disability**	
No	17 (51.5)
Yes	9 (27.3)
Prefer not to say	4 (12.1)
Missing	3 (9.1)
**Highest level of education**	
High school	16 (48.5)
College	11 (33.3)
Postgraduate	1 (3.0)
Other	3 (9.1)
Missing	2 (6.1)
**Housing situation**	
Homeowner	18 (54.6)
Rent (private landlord)	1 (3.0)
Rent (social housing)	8 (24.2)
Live with parents	4 (12.1)
House/flat share	0 (0.0)
Other	0 (0.0)
Missing	2 (6.1)
**Income**	
£0-£14,999	10 (30.3)
£15,000–24,999	2 (6.1)
£25,000-£34,999	0 (0.0)
£35,000+	0 (0.0)
Missing	21 (63.6)
**Health status**	
Very good	3 (9.1)
Good	11 (33.3)
Fair	10 (30.3)
Bad	5 (15.2)
Do not wish to say	4 (12.1)

Note: ^1^percentage vary due to missing data, ^2^includes a combination of being unemployed and unable to work for physical and mental needs (6.1%), being unable to work due to physical and mental needs (9.1%) being unemployed and a homeowner (3.0%), employ

### Observations

Sessions took place in community houses and church kitchens, which were well-equipped and allowed for group cooking and shared dining. The overall setting was informal, welcoming, and inclusive. The layout supported collaboration and social engagement, with shared preparation areas and seating. The environment contributed positively to participants’ comfort and willingness to engage.

Participants were primarily engaged in cooking activities throughout the sessions. All individuals arrived expecting to cook, and this formed the central structure of the session. Although the cooking activities sometimes implicitly encouraged the use of leftovers or portion control, these elements were not explicitly framed in terms of reducing household food waste (HHFW), nor were topics like meal planning or the use of shopping lists addressed in any structured way. In two of the observed kitchens, the theme of food waste reduction was not mentioned at all. Where it was mentioned, it was discussed only briefly and not in depth.

Interactions between participants, volunteers, and staff were generally relaxed and supportive. Participants worked together in small teams or pairs, often sharing responsibilities and offering assistance to one another. While most conversations remained light-hearted and centred around the cooking tasks, some deeper or personal discussions occurred during natural breaks, such as when food was in the oven. However, these were infrequent. Some individuals were more socially dominant, while others preferred to observe rather than actively contribute to discussions.

A range of kitchen tools and materials were used, including measuring cups, recipe books, and standard cooking equipment. Some of these items, such as WRAP recommended portioning tools, were available but not central to the session or actively used for educational purposes. Printed materials related to WRAP and LFHW were not observed in use during any of the sessions. This indicates that while some WRAP-aligned objects were present, they were not used in a way that supported behaviour change education around HHFW.

Participants included adults of varying ages and abilities, including individuals with cognitive or physical impairments. Volunteers and Borough Council staff coordinated the sessions and supported inclusive engagement. Participants were observed to value the opportunity to take part in a structured, social activity. Their roles in the kitchen were flexible, allowing people to contribute according to their comfort and capability. Values observed included cooperation, mutual support, and enjoyment of shared meals, though participants generally did not express or demonstrate an explicit focus on reducing food waste.

### Interviews

A total of 19 interviews were conducted with individual community kitchen (*n* = 14) attendees, volunteers (*n* = 3) and Borough Council staff (*n* = 2). Of the attendees, ten were women and 4 men. For those of whom we have complete data on age (*n* = 9), age ranged between 25 and 74 years with the majority being > 55 (*n* = 7). For those with data for employment status (*n* = 11), five were retired, three were unable to work due to physical or mental disabilities, one was unemployed and two were employed.

Thematic analysis resulted in two primary themes: Reasons for, and experiences of, attending and health and wellbeing benefits.

#### Reasons for, and experiences of, attending

Overall, staff, volunteers, and attendees reported that they enjoyed being involved with the community kitchens perceiving them to be a good idea, valuable and worthwhile:

*“Oh*,* I think it’s really good. I think it’s a really good idea. I love it” P3.*

*“There’s a nice bunch of people*,* it’s really good” P14.*

Reasons for attending the sessions included them being a source of social interaction and company, offering structure and routine for attendees who lived alone and offering an opportunity for attendees to have time to themselves, time without a carer, learn new cooking skills and have a break from other responsibilities (e.g., caring):

*“You know I live on my own*,* so you’ve got nobody to chat to all the time*,* so it is a one day a week and you know you’re going to be chatting all the time” P4.*

*“90% of the reason they’re there is obviously for companionship and to meet other “people” S/V19.*

*“It’s given me a new structure to like because I think that is what we all lack. We all. I think we all thrive on doing things that are a set pattern and set way. We’re all creatures of habits aren’t we really?” P7.*

Staff, volunteers, and attendees all reported that they enjoyed the sessions, some reporting them to be the highlight of their week, but that the activity (i.e., cooking) was not central to their enjoyment. Rather, the opportunity to socialise was most commonly described as the main reason for and the aspect most enjoyed by attendees. The session activity (i.e., cooking) was reported to be a bonus of attendance:

*“it’s a social circle*,* an opportunity to have a coffee or and just a general chat. That’s the main benefit for me” P11.*

*“Otherwise*,* you’re quite isolated. You know when you spend so much time indoors. It is quite nice to have other women to talk to” P17.*

*“I think sometimes the socialising has become more important” P7.*

The location of the community kitchen was reported to be a barrier to attendance and social interaction due to physical impairments and/or lack of public transport:

*“The biggest hurdle you’ve got is probably location” S/V19*.

*“Well*,* I don’t drive. I gave up driving*,* so I don’t drive anymore*,* haven’t done so for years but um no I can’t get to anywhere*,* the bus is only every hour and a half*,* it’s difficult to get out of the village” P2.*

*“You know I can only get the bus to a certain place then I’ve got to get either another Taxi or walk up*,* which for me just takes ages and I’ll be exhausted by the time I got there” P11.*

#### Health and well-being benefits

Staff, volunteers, and attendees perceived a range of benefits from attending the community kitchen sessions, for themselves and other particular groups of individuals such as those living alone:

*“Because I do live on my own. That is why a lot of us go actually” P2.*

*“Most of them (attend the community kitchens) because they’re on their own” S/V1.*

For example, learning new cooking skills and trying new foods were frequently reported as was increasing knowledge about nutrition and learning to be healthier. Attending the community kitchens was also reported to be associated with increased confidence, more frequent social interaction, increased physical activity, and feelings of mutual support:

*“I mean*,* I used to be ever so shy*,* I wouldn’t mix you know*,* couldn’t face people*,* but now I’ve joined this (community kitchen) I seem more open and I’m getting to interact with different people” P12.*

*“When we’re there*,* when we’re cooking*,* we all more or less support each other and we will help each other*,* you know in whatever way we can” P11.*

These impacts were particularly felt by attendees with a cognitive or physical impairment (e.g., anxiety) and attendees who were widowed, lived alone, or had retired and were more isolated from everyday social interactions because they were no longer in employment:

*“It just gave me more confidence and stuff. Because obviously I suffer with anxiety and it’s helped me*,* […] make friends and talk to people more and interact with people” P3.*

*“I would say people are benefiting the most are the people that perhaps are not as able” P10.*

Participants also reported that their involvement in the community kitchens had resulted in new friendships. These friendships were however, largely confined to the sessions with interactions beyond the sessions rarely occurring:

*“I see my friend. I see another couple of girls occasionally*,* but no*,* we just meet up on this Wednesday thing (when the community kitchens occur)” P6.*

The social contact, friendship and supportive environment offered by the community kitchens was consequently reported to be missed during the school holidays when the community kitchens sessions stopped.

“*We miss it when the holidays are on*,* and we don’t meet*” P2.

The health and wellbeing impacts reported by attendees were described as part of broader community engagement rather than being solely attributed to kitchens:

*“I can’t say it’s the kitchen that’s impacted my everyday life” P11.*

*“I’m thinking it started (growth in confidence) when they started going down to the church on Wednesday” P14.*

## Discussion

The main finding of this research suggests that social interaction to be the primary benefit experienced by individuals attending the community kitchen scheme in Leicestershire. Findings therefore suggest a possible gap between the reasons behind the development and implementation of the community kitchen scheme, their intended impact and the actual practices and benefits experienced by attendees. While the community kitchens were developed to reduce HHFW, reducing HHFW did not appear to be the core element of the community kitchens, nor the reason for why people chose to attend. Notwithstanding this, the community kitchens were considered a valued and worthwhile intervention by all those involved with the scheme, particularly in terms of the kitchens offering opportunities to socialise in an inclusive supportive environment. In line with previous research [14] the primary reason for attendance was reported to be increased social interaction. The findings indicate that the focus of the community kitchens may have shifted from the original goal of reducing household food waste to promoting social benefits, particularly enhancing social interaction and community connectedness. This shift aligns with evidence suggesting that community food programs often serve as important social hubs that facilitate community networks and improve cooking skills, which in turn can contribute to improved wellbeing and food-related confidence [41]. Social engagement through shared cooking activities has been shown to foster supportive relationships and increase motivation to adopt healthier food practices [42]. Recognizing and embracing the social function of community kitchens may therefore enhance their reach and perceived value, while targeted efforts can be incorporated to maintain some focus on reducing food waste.

The community kitchens scheme is therefore suggested to be a possible intervention for reducing the risk factors associated with social isolation (e.g., reduced wellbeing, mortality, depression, cognitive decline [43–45]). However, results also suggest that they may have limited impact in supporting longer-term or deeper relationships beyond the sessions, indicating a need for additional opportunities for connections between attendees such as follow up groups or partner programmes with other community organisations to better address loneliness in addition to social isolation.

These findings are also important when considering the population these community kitchens appear to attract because most of the participants were older adults, a population group at higher risk of social isolation and loneliness because of decreased economic and social resources, functional limitations, and the death of significant others [45]. The community kitchens were also reported to be beneficial for individuals of varying levels of ability with and without specific needs for improving social interaction, along with the co-benefits of education and an improved sense of confidence, and general mental health. The community kitchens offered attendees a safe environment to engage in a collaborative activity with others which contributed to the creation of an inclusive environment that facilitated and promoted social interaction and support. This reinforces previous research that suggests effective interventions to be those that offer a social activity and/or support within a group format [23]. Community kitchens were therefore also suggested to be an important health intervention for individuals who are at greater risk of experiencing health inequalities (e.g., individuals with learning disabilities). Given these benefits, policy makers and community organisations should consider community kitchens as a multi-dimensional public health intervention that not only supports social wellbeing but also contributes to reducing health disparities in vulnerable groups. Expanding the model to include tailored sessions for specific needs (e.g., mental health, disability, older adults) could enhance reach and effectiveness.

While attendance at the community kitchen was associated with the health and wellbeing benefits of social interaction, the health and wellbeing benefits were reported to operate within a broader context. For example, participants reported that attending other community activities had encouraged their attendance at the community kitchen and vice versa. improvements in social interaction and the perceived health and wellbeing benefits experienced are therefore suggested to be the result of a combination of activities and engagement with the community rather than being specifically from attending the community kitchens alone. This suggests the importance of embedding community kitchens within a wider network of community services and programmes to amplify their impact. Strengthening links and referrals between services may improve participant engagement and enhance the cumulative benefits of social participation.

Further, barriers were reported to impact participation, with kitchen location and accessibility being disclosed to be problematic, particularly for those with physical or cognitive needs. The community kitchens were initially established to offer all individuals with varying needs and levels of cooking ability a place where they could learn how to reduce HHFW. While the results show the community kitchens to be particularly beneficial to individuals with varying levels of social, cognitive, and physical needs, the barriers reported highlight the importance of future community kitchens needing to consider the population they wish to serve when deciding location and access requirements. This is particularly important if the community kitchens are to be effective in offering individuals at risk of social isolation the opportunity to participate in a collaborative activity and experience the associated health and wellbeing benefits. Barriers related to location and accessibility are common challenges in community-based interventions. Selecting venues that are physically accessible, located near public transport, and familiar to the target population can enhance participation and engagement [46]. In addition, flexible scheduling and consideration of participants’ routines and responsibilities are important factors to improve attendance and inclusivity [47]. These considerations should be integrated into the planning of community kitchens to maximize their reach and effectiveness.

Although reducing HHFW was the original aim of the intervention, the findings suggest this was not a key focus in practice. Previous interventions that have successfully reduced HHFW, such as structured WRAP programmes, community-based food waste challenges, and targeted cooking workshops, have typically included specific educational content, behaviour change techniques (e.g., goal setting, reminders), and visible prompts that support learning around meal planning, portion control, and food storage (e.g., [30, 48]). To improve alignment with these goals, community kitchens could incorporate a stronger educational component, with structured discussions or demonstrations linked to WRAP and LFHW resources and integrate simple tracking tools or prompts to reinforce behaviour change outside the session. In doing so, community kitchens could potentially maintain their social value while also becoming more effective at addressing food waste reduction, thereby achieving a dual public health and environmental impact.

To enhance accessibility, future community kitchen initiatives should conduct community needs assessments and incorporate inclusive design principles in their planning. This may include selecting venues with appropriate physical access, offering transportation support and scheduling activities at times that accommodate participants with varied routines and responsibilities.

Notably, triangulation of observational and interview data revealed that while HHFW-reducing practices were present during sessions, participants did not consistently identify these as key benefits or outcomes. This disconnect suggests that although HHFW content is embedded in activities, it may not be sufficiently emphasised or internalized by attendees. To address this gap, future community kitchens could increase explicit focus on food waste reduction through facilitated discussions, clear goal setting, and more prominent use of WRAP and LFHW resources, thereby strengthening participant awareness and motivation to reduce household food waste.

### Strengths and limitations

Location of the community kitchen sessions may have had an impact on recruitment for surveys and interviews and thus the generalisability of the results to all participants attending the community kitchens. The researcher was unable to actively attend the community kitchens on a regular basis and recruit attendees due to the proximity of the kitchens. More attendees may have been recruited if the researcher had had more first-hand interactions with the attendees. Moreover, the researcher may have been able to assist with recruiting attendees less likely to actively volunteer to participate (e.g., attendees who were not verbal), increasing the sample representativeness. Although the sample captured a range of experiences, additional perspectives and benefits may have been reported by other demographics. Increasing the researcher’s presence at the community kitchens may also have created further opportunities or alternative methods of participation for attendees who were not verbal (e.g., written interview). Conversely, the researcher’s presence during the observations may have indirectly influenced the participants behaviour, the way they interacted and communicated with one another, the sessions activity, and the way the session was planned and facilitated by volunteers and/or Borough Council staff. Notwithstanding these limitations, strengths include the mixed methods approach using questionnaires, observations, and interviews, which helped to provide a more in depth understanding of complex phenomena. However, as data relied on self-reported experiences, there is a possibility of social desirability or recall bias. The cross-sectional design also limits the ability to infer causality or long-term outcomes. In addition, the co-production of the study design, methods, and materials with representatives of the public and relevant stakeholders from the start and throughout the duration of the study benefitted the inclusivity, accessibility and relevance of the study materials and methods, ensuring that we were able to achieve the research aims.

## Conclusion

The community kitchens were perceived by participants to have a positive impact on social isolation despite their original aim being to reduce HHFW. Although HHFW did not remain the main focus of the community kitchens in practice, the kitchens continue to be a valued community-based intervention. A refocus of advertising the community kitchens social and wellbeing benefits may help attract more participants and drive the expansion of the community kitchen scheme across Leicestershire and other geographies. However, further research with larger and more diverse samples is needed to examine the extent and sustainability of these perceived benefits and to explore how best to integrate food waste reduction goals.

## Supplementary Information

Supplementary Material 1.

## Data Availability

Anonymised survey data, qualitative transcripts, and observation fieldnotes, used during the current study are available from the first author on reasonable request.

## Abbreviations

HHFW: Household food waste
WRAP: Waste and Resources Action Programme
LCC: Leicestershire County Council
LFHW: Love food hate waste
