# Supplementary material for: A qualitative exploration of community kitchens to reduce household food waste as a public health intervention
Source: BMC Public Health. 2025 Dec 10;26:183. doi: 10.1186/s12889-025-25903-2 (PMC12801577; doi:10.1186/s12889-025-25903-2)
Supplement: Supplementary file 1 — Supplementary Material 1. [file 12889_2025_25903_MOESM1_ESM.docx]

Supplementary material 1: Mixed method survey

**Evaluating the Leicestershire Community Kitchen Scheme**

**Mixed method survey**

The following questions ask about you, your health and wellbeing and your experiences of the community kitchens. There are no right or wrong answers. If you are not clear about a particular question, have any questions or would like help completing the survey, please contact China ([china.harrison@bristol.ac.uk](mailto:china.harrison@bristol.ac.uk), 07790 773945), one of the community kitchen volunteers or ask Rachel, Shirley or Melitza from Hinckley and Bosworth Borough Council.

**Section 1: Routine attendance data**

Please tell us which community kitchen you attend/ed

- Name of community kitchen

Please tell us in years and/or months, how long you have been attending the community kitchen

Years:_____ Months_____

If applicable, please tell us what month and year you stopped attending the community kitchen

Months_____ Year:_____

Please tell us how regularly you attend/ed the community kitchen

- Weekly
- Fortnightly
- Monthly
- Other: please specify___________________

Please would you tell us, in your own words, why you started participating in the community kitchen?

____________________________open text____________________________________

If applicable, please would you tell us, in your own words, why you stopped participating in the community kitchen?

____________________________open text____________________________________

**Section 2: Demographics**

What is your gender?

- Male
- Female
- Other
- Prefer not to say

What are the first four characters of the postcode of your current address?

_____________________(open text)_________________

Please tell us your age:

- Under 18
- 18-24
- 25-34
- 35-55
- 45-54
- 55-64
- 65-74
- 75-84
- 85+

The Equality Act (2010) defines a person as having a disability if he or she ‘has a physical or mental impairment that has a ‘substantial’ and ‘long term’ negative effect on your ability to do normal daily activities’. Do you consider yourself to have such a disability?

- Yes
- No
- Do not wish to say

**Section 3: Socioeconomics and food security**

What is the highest level of education you have completed

- Did not attend school
- High school
- College
- Undergraduate degree
- Postgraduate
- Other (please specify) __________________

What is your current work situation?

- Employed full time
- Employed part time
- Self-employed
- Unemployed
- Student
- Retired
- Unable to work (physical health)
- Unable to work (mental health)
- Unable to work (carer)
- Home maker

What us your approximate average household income?

- £0-£14,999
- £15,000-£24,999
- £25,000-£34,999
- £35,000+

What is your current housing situation?

- Own my own home
- Rent from a private landlord
- Rent through social housing
- Live with parents
- House/flat share

How strongly do you agree or disagree with the following statements? (5-point Likert scale; strongly disagree, slightly disagree, neutral, slightly agree, strongly agree)

- In the last year, me and/or my family has had enough of the kinds of food we want to eat
- I feel that I can easily buy healthy food in my neighbourhood
- In the last year I have worried that the food I have at home would run out before I had money to buy more
- In the last year, I, or someone else living with me, has cut the size of our meals, or skipped meals because there was not enough money for food
- In the last year I have been hungry but did not eat because there was not enough money for food
- I feel I have access to a kitchen and the things I need to cook with to make meals for me and other members of my household
- I feel confident preparing and cooking meals using fresh ingredients
- I often spend less on food to be able to afford other things
- I feel that myself and/or people I live with often do not have enough money to buy the food we need

If you feel you often don’t have enough money to buy food, please tell us why you think that is

______________(open text box)____________________________________________

**Section 4:** **health and wellbeing (physical, mental and social)**

**General Health status (SHH)**

Please tell us how your health is in general. Would you say it is:

- Very good
- Good
- Fair
- Bad
- Very bad
- Don’t wish to say

**Mental health status (MHI-5)***

Please read each question and tick the box that best describes how things have been FOR YOU during the past month using the 6-point scale (all the time, most of the time, a good bit of time, some of the time, a little of the time, none of the time). There are no right or wrong answers.

- During the past month, how much of the time were you a happy person?
- How much of the time, during the past month, have you felt calm and peaceful?
- How much of the time, during the past month, have you been a very nervous person?
- How much of the time, during the past month, have you felt downhearted and blue?
- How much of the time, during the past month, have you felt so down in the dumps that nothing could cheer you up?

**Social support and connectedness (DSSI-10)**

**Social interaction***

1. Other than members of your family, how many people in your local area do you feel you can depend on or feel close to? (Scoring: None (1), 1-2 people (2), more than 2 people (3))

2. How many times during the past week did you spend time with someone who does not live with you? That is, you went to see them, or they came to visit you, or you went out together? (Scoring: None (1), once (2), twice (2), three times (3), four times (3), five times (3), six times (3), seven or more times (3)).

3. Number of times in past week talked with friends/relatives on the telephone in the past week (either they called your, or you called them)? (Scoring: None (1), once (1), twice (2), three times (2), four times (2), five times (2), six times (3), seven or more times (3)).

4. Number of times in the past week attended meetings of clubs, religious groups, or other groups that you belong to (other than work) (Scoring: None (1), once (1), twice (2), three times (2), four times (2), five times (2), six times (3), seven or more times (3)).

*Sum the scores for items 1-4. The scale ranges from 4-12 with higher scores indicating more social interaction. There is no imputation for missing items.

**Social satisfaction***

1. Does it seem that your family and friends (people who are important to you) understand you? (Scoring: Hardly ever (1), some of the time (2), most of the time (3))

2. Do you feel useful to family and friends (people who are important to you)? (Scoring: Hardly ever (1), some of the time (2), most of the time (3))

3. Do you know what’s happening/going on with family and friends? (Scoring: Hardly ever (1), some of the time (2), most of the time (3))

4. Do you feel you have a definite role in the family and among your friends? (Scoring: Hardly ever (1), some of the time (2), most of the time (3))

5. When you are talking with your friends and family, do you feel you are being listened to? (Scoring: Hardly ever (1), some of the time (2), most of the time (3))

6.Can you talk about your deepest problems with at least some of your family and friends? (Scoring: Hardly ever (1), some of the time (2), most of the time (3))

*Sum of codes with higher scores indicating more social support. There is no imputation for missing items.

A score for Duke social support index is calculated as the sum of 10 items with mean imputation for up to two missing items.

**Section 5: Aims and impact of community kitchens**

Please tell us, in your own words, what you think is the purpose of the community kitchens?

___________________________________open text___________________________­­­­­

Has anything changed for you since you started attending the community kitchens?

- Yes
- No

If yes, please tell us, in your own words, what has changed and how.

___________________________________open text___________________________­­­­­

Please tell us, in your own words, what you consider to be the advantages or benefits of attending the community kitchen

___________________________________open text___________________________­­­­­

Please tell us, in your own words, what you consider to be (or could be) the disadvantages of attending the community kitchen for you or for others

___________________________________open text___________________________

The researchers from the University of Bristol would like to talk to individuals about their experiences of attending community kitchens. If you are interested in participating in a short interview, please leave your email address of phone number so that one of the researchers can contact you with more information and details about how to participate. Those who choose to participate in the interview will be given a £15 voucher as a thank you for their participation. _

Email__________________(open text)_____________________________

_Phone number _________ _(open text)_____________________________

Supplementary material 2: Observation framework

**Observation framework**

| Date: | Project name: | Community kitchen: |
| --- | --- | --- |
| Time: | Researcher name: |  |

| **Activities**  *What actions and behaviours are people engaged in? what are the modes people work in, and the specific activities and processes they go through?* | **Environments**  *What is the overall setting in which the activities are taking place?*  *What is the character and function of the space overall, of each individual’s space and shared spaces? How are people behaving in the environment?* | **Interactions**  *What are the basic interactions occurring between a person and someone or something else? What is the nature of routine and interactions between people and between people and objects?*  *What effect do people have on activities and environment?* | **Objects**  *What are the details that form the environment? What are the items the people are interacting with? How do objects relate to people,* *activities, and interactions? How do the objects relate to the activities?* | **Users**  Who are the people being observed? What are their personal qualities? How do they engage with other people? What are their roles and relationships? What are their values and predispositions? |
| --- | --- | --- | --- | --- |
|  |  |  |  |  |

Supplementary material 3: Interview topic guide

**Evaluating the community kitchen scheme**

**Topic guide for Semi-structured Interview: community kitchen participants**

*Note:*

*This topic guide is indicative. It is a guide to the topics to be covered during the interviews (and not a script) therefore the order of topics, and the precise* *way they are addressed, will be flexible according to the circumstances.*

*Writing in italics does not form part of the interview and is for researcher purposes only.*

**Sample introduction**

- Thank you for agreeing to participate
- Introduce background information about CKs and potential impact
- Recordings, confidentiality, and anonymity
- Any questions

**Ice breaker questions** *(data on who is attending and contextual influences)*

1. Would you tell us a bit about yourself and how you heard about the community kitchens?
2. What made you decide to start attending the community kitchens?
3. Did you know someone who was already attending?
4. How was the community kitchen advertised – was it for a particular group (e.g., bereaved men)

**What is happening during** community kitchens *(data on contextual influences and participant experiences)*

1. Please would you talk us through what happens during a typical community kitchen session?
2. Please would you tell us what you think of the community kitchens s
3. What have your experiences of attending been so far?
4. What have you enjoyed?
5. What have you not enjoyed?
6. What have you found to be useful?
7. Is there a particular element of the community kitchen session that you like?

**What is happening during** community kitchens **s** *(data on social interactions)*

1. How do you feel participating in activities with other individuals?
2. How supported do you feel during the community kitchen session?
3. How supported do you feel when you are not at the community kitchen?
4. What types of discussions happen during community kitchens?
5. what is your role in these discussions?
6. How do you think participation in the community kitchens has influenced the way you interact and socialise with other people?
7. Do you socialise more?
8. Do you feel comfortable in social situations?

**Impact of** community kitchen on **everyday life** *(perceived impacts, mechanisms of change,* *advantages, and disadvantages of CK)*

1. How do you think participating in the community kitchen has influenced your everyday life?
2. In what way?
3. Could you tell us a little bit more about this?
4. What, if any, new things have you learnt from attending community kitchens?
5. Could you tell us a little bit more about that?
6. How has what you have learnt transferred into your everyday life?
7. Overall, what impact do you think participating in community kitchen s has had on you?
8. Why do you think this is?
9. What have you found to be the most helpful?
10. What have you found to be the least helpful?
11. Have there been any distinct changes in your life since you started participating in the community kitchen.
12. Please would you tell me a little bit more about this?
13. What benefits, if any, would people gain from attending the community kitchen s?
14. Do you foresee any potential harm or negatives from attending the community kitchen s?
15. Is there anything about the community kitchen s that you would improve?
16. Could you tell us a little bit more about this?
17. Is there anything else you would like to mention about your participation in the community kitchens?
18. Do you have any other feedback or comments you would like to share?
